# The Role of PET Detection of Biomarkers in Early Diagnosis, Progression, and Prognosis of Alzheimer’s Disease: A Systematic Review

**DOI:** 10.7759/cureus.77781

**Published:** 2025-01-21

**Authors:** Viktoriia Zarovniaeva, Summayya Anwar, Saba Kazmi, Kimberly Cortez Perez, Sehej Sandhu, Lubna Mohammed

**Affiliations:** 1 Internal Medicine, California Institute of Behavioral Neurosciences & Psychology, Fairfield, USA; 2 Biosciences, COMSATS University Islamabad, Islamabad, PAK

**Keywords:** alzheimer, alzheimer's disease, amyloid pet, amyloid plaques, imaging biomarker, pet ct scan, tau protein

## Abstract

Alzheimer’s disease (AD) is a chronic neurologic disease characterized by the deposition of Aβ amyloid and tau protein in the neural tissue, which leads to gradual and irreversible deterioration of memory. Positron emission tomography (PET) showed high potential in diagnosing AD. It provided a unique opportunity to assess cerebral amyloid plaques and tau neurofibrillary tangle deposits in the brain tissue without invasive procedures in vivo. Many studies have been focused on PET diagnosis of AD in recent years, which has significantly improved diagnosis and treatment strategies. This review study aims to summarize the role and emphasize the benefits of PET detection of AD biomarkers in early stages, clinical and histological progression assessment, and predicting AD outcomes. Relevant articles published in the last five years, from September 1, 2019, to October 30, 2024, were searched through authentic databases such as PubMed, PubMed Central, Europe PubMed Central, Science Direct, Cochrane Library, and Google Scholar. In this systematic review, we included articles published in English, with available full text, based on human trials, with relevant information regarding participants who underwent PET of the brain to diagnose AD biomarkers. The study strictly followed the Preferred Reporting Items for Systematic Reviews and Meta-Analyses (PRISMA) 2020 guidelines and recommendations. The Joanna Briggs Institute (JBI) critical appraisal methods were used to evaluate all selected cross-sectional research, and the Newcastle-Ottawa Scale (NOS) was used to assess the cohort and longitudinal studies. Eleven relevant articles were included in this systematic review, and 2,203 males and females participated. The study revealed that the detection of beta-amyloid PET showed high-precious results in early diagnosis of AD. The detection of tau protein showed a high potential for estimation of the clinical and histological progression and prognosis of AD in longitudinal studies. Identifying amyloid and tau protein accumulation and glucose metabolism alterations is highly predictive of neurodegeneration in preclinical and mild cognitive impairment stages.

## Introduction and background

Alzheimer’s disease (AD) is a common chronic neurologic disease leading to gradual deterioration of episodic memory, cognitive impairment, behavioral change, reduced mobility, hallucinations, and seizures [1]. Currently, 6.9 million Americans aged 65 years and older are thought to have AD [2]. About 50% to 75% of cases of dementia are caused by AD [1]. In 2021, statistically, 119,399 lethal cases of AD were reported officially. In recent years, official counts have still been compiled. For Americans aged 65 years and older, AD continues to rank as the sixth most common cause of death, and recorded mortality from AD has risen by more than 140% in recent decades [2].

Pathogenesis of AD is related to degenerative pathological changes in the neural tissue associated with the deposition of beta-amyloid plaques and phosphorylated tau proteins. Detection of these biomarkers, depending on the accumulation quantity, confirms a diagnosis and helps distinguish between dementias. Advanced age is the most essential risk factor for AD development [3]. The etiology of AD is closely associated with *APOE* alleles, and the genetic risk of disease development is around 60-80% in patients with that gene [4,5]. In familiar forms of AD, autosomal dominant gene mutations of the presenilin 2 genes on chromosome 1, the amyloid precursor protein gene on chromosome 21, and the presenilin 1 gene on chromosome 14 can lead to the early onset of AD in the atypical type, which is about 1% to 2% of AD cases [6]. Late-onset AD has a polygenic etiology [7].

AD’s preclinical stage spans 10-20 years and is marked by progressive cognitive decline accompanied by episodic memory loss, difficulty recalling recent information, and impairment of information storage [1,7]. In the early stages, physical examination is unremarkable or reveals mild cognitive decline. According to disease progression, extrapyramidal symptoms may be present [7]. In advanced stages, the disease affects daily life and leads to severe disability [1,7]. Therefore, diagnosis in the early stages and differential diagnosis between other dementias are essential for appropriate patient management and prescribing relevant treatment.

In recent years, diagnosis using analysis of fluid biomarkers and imaging methods has significantly improved the early diagnosis of AD. The National Institute on Aging and Alzheimer’s Association (NIA-AA) conducted a research study. In their list of proven and validated biomarkers of AD, they included the detection of Aβ-amyloid and tau-protein deposits on positron emission tomography (PET), the cerebrospinal fluid (CSF) concentration of Aβ42, the ratio of Aβ42 to Aβ40, and the levels of total tau (t-tau) and p-tau 181 in the CSF [8].

Radiological methods of diagnosing AD have been actively involved in clinical studies since the 1980s [9]. Despite the radiation exposure, PET is the most informative radiological method and a golden standard for diagnosing AD. That radiological method allows for assessing cerebral amyloid plaques and tau neurofibrillary tangle deposits in the brain tissue without invasive procedures in vivo [10]. PET estimates standardized uptake value ratios (SUVRs) of radiotracers such as 18F-florbetaben, [11C] PiB, and 18F-THK5351, which bind Aβ-amyloid and tau-protein [11].

PET tau-protein detection demonstrated significant correlations with cognitive performances and showed perspectives to explain cognitive decline [10]. Histopathological assessment of brain tissue in vitro validated visual PET imagination of amyloid deposition and the positive correlation between tau-protein accumulation and cognitive functions with high accuracy [10,11]. Additionally, tau PET imaging is an informative tool for estimating the effects of disease-modifying therapeutic strategies in AD by facilitating the evaluation of the deposition of neurofibrillary tangles of tau pathology in longitudinal studies [12]. Compared to magnetic resonance imaging (MRI), one of the noninvasive and safe radiological methods that estimate the volume of the hippocampus and the severity of cortical atrophy, PET detection of AD biomarkers is more specific. Volumetric MRI of the hippocampus showed low specificity in the differential diagnosis between other degenerative diseases and other causes of dementia [9].

**Figure 1:**
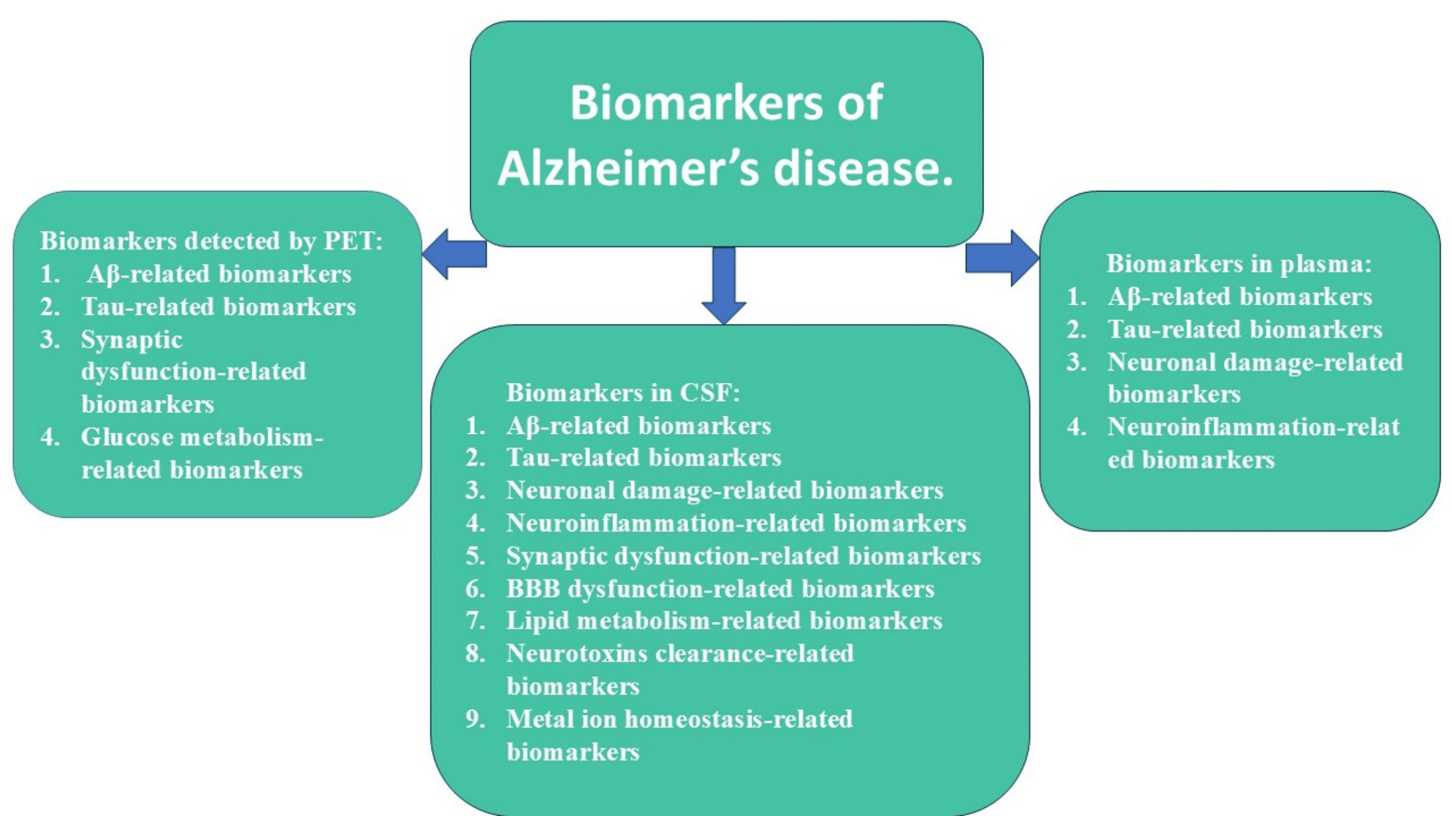
Alzheimer’s disease biomarkers. BBB: blood-brain barrier.

In recent years, the detection of AD biomarkers in CSF has improved diagnostic accuracy, including the preclinical stage. The method is sensitive and allows for the differentiation of AD and other psychiatric disorders, including the early stages. In individuals with unclear clinical dementia diagnosis and patients with combined brain pathology, such as AD and cerebrovascular illness, the CSF biomarkers detection is highly specific [13]. However, detecting AD biomarkers in CSF demonstrates only snapshots of specific biomarkers without a detailed perspective and information about particular affected brain regions. Compared to the detection of the biomarkers in CSF, PET detection of AD biomarkers is a noninvasive method that assesses Aβ-amyloid and tau-protein distribution in the whole brain tissue and progression in dynamic in a longitudinal perspective, which would be beneficial for the prediction of clinical progression of disease and treatment assessment. Apart from the above, there are blood-based biomarkers for diagnosing AD, which have not yet been approved. However, detecting biomarkers in the plasma correlates with pathological and clinical presentation and can predict the future development of AD [5]. All potential biomarkers are demonstrated in Figure 1.

However, the potential of PET imaging of AD biomarkers has not been fully utilized, and many unanswered questions remain. Recent advances in AD research suggest that visual evaluation and global semi-quantitative metrics, like the SUVR, may not be the best options for AD diagnosis in patients cognitively unimpaired (CU) and mild cognitive impairment (MCI) patients due to false positive results [14]. Several recent studies suggest that Aβ-amyloid detection is predictable only in the early stages of the disease and switches to a plateau in the advanced stages. Apart from the above, some longitudinal studies showed discordance between PET and CSF biomarkers assessments in advanced stages of disease [15]. Additionally, in recent years, many studies have revealed the promising potential of monoclonal anti‐Aβ antibodies for disease progress-changing treatment of AD, which requires adequate evaluation of treatment effect and disease clinical progression [16]. Effective early diagnosis of AD and timely prescription of appropriate treatment is essential for patient prognosis and survival. It also allows patients and their caregivers to strategize for the future and implement lifestyle modifications that could extend their quality of life [17]. Therefore, future studies on early diagnosis and assessing AD progression are necessary to estimate which biomarker and method of diagnosis is more relevant. This systematic review identifies gaps in PET imaging for AD. The aim of this study is to explore and summarize the role and emphasize the benefits of PET detection of AD biomarkers in early diagnosis, clinical and histological progression assessment, and AD outcomes to improve the disease's diagnosis and evaluate the effects of treatment and management strategies.

## Review

Methods

To create this systematic review, we preciously followed the Preferred Reporting Items for Systematic Reviews and Meta-Analyses (PRISMA) 2020 guidelines [18].

Database and Search Strategy

To find relevant articles, we performed a literature search from September 1, 2019, to October 30, 2024, through multiple databases such as PubMed using PubMed Medical Subject Headings (MeSH), PubMed Central, Europe PubMed Central, Science Direct, Cochrane Library, and Google Scholar. Keywords such as Alzheimer’s disease, positron emission tomography/computed tomography (PET-CT), biomarkers, amyloid, tau proteins, prognosis, progression, and diagnosis were entered into search engines and used to create our search strategy. Additionally, we performed manual citation searching. Each co-author actively participated in the creation of this systematic review, contributing to the data collection and selection process and quality appraisal of selected articles. Additionally, co-authors assessed this article's content and structure and checked for grammatical mistakes. We conducted Zoom meetings every week, during which all co-authors discussed their updates and proposed new suggestions, assessment results, and findings. More detailed search strategies and identified articles are described in Table 1.

**Table 1:** Articles identified using each database. MeSH: Medical Subject Headings; PMC: PubMed Central.

Database	Keywords	Search strategy	Filters	Search result
PubMed MeSH/Medline/PMC	Alzheimer’s disease, PET-CT, amyloid, biomarkers, prognosis, disease progression diagnosis, early diagnosis	(((("Alzheimer Disease/diagnosis"[Majr] OR "Alzheimer Disease/diagnostic imaging"[Majr] OR "Alzheimer Disease/etiology"[Majr] OR "Alzheimer Disease/pathology"[Majr] OR "Alzheimer Disease/therapy"[Majr]) AND ("Positron Emission Tomography Computed Tomography/methods"[Majr])) AND ("Disease Progression"[Majr] OR "Prognosis"[Majr]) OR "Diagnosis"[Majr] OR "Early Diagnosis"[Majr]) AND ("Biomarkers"[Majr])) AND ("Amyloid/metabolism"[Majr])	Last five years, humans, English language, exclude preprints	47
PubMed MeSH. Advanced strategy/Medline/PMC	Alzheimer’s disease, PET-CT, plaque, amyloid, biomarkers, prognosis, disease progression diagnosis, early diagnosis	(((((( "Alzheimer Disease/diagnosis"[Majr] OR "Alzheimer Disease/diagnostic imaging"[Majr] OR "Alzheimer Disease/etiology"[Majr] OR "Alzheimer Disease/pathology"[Majr] OR "Alzheimer Disease/therapy"[Majr] ) OR Alzheimer’s disease OR Dementia) AND ("Positron Emission Tomography Computed Tomography/methods"[Majr] OR PET CT OR Neuroimaging)) AND ("Disease Progression"[Majr] OR Disease progression OR Disease deterioration)) AND ("Prognosis"[Majr] OR Prognosis OR outcomes)) AND (“Diagnosis”[Majr] OR “Early diagnosis”[Majr] OR Diagnosis OR Early diagnosis)) AND ("Biomarkers"[Majr] OR Biomarkers) AND ("Amyloid/metabolism"[Majr] OR Amyloid)	Last five years, humans, English language, exclude preprints	59
PubMed/Medline/PMC	Alzheimer’s disease, PET CT	(PET CT) AND (Alzheimer's disease)	Last five years, humans, English language, exclude preprints	376
PubMed Central advanced	Alzheimer’s disease, PET CT	(((PET CT[Abstract]) OR PET CT[Title])) AND ((Alzheimer's disease[Abstract]) OR Alzheimer's disease[Title])	Last five years	71
PubMed Central	Alzheimer’s disease, PET CT, biomarkers, prognosis, disease progression, amyloid, tau protein, diagnosis, plaque	Alzheimer's disease AND PET CT AND Biomarkers AND prognosis AND disease progression AND Amyloid AND Tau protein AND plaque, amyloid AND diagnosis	Last five years	385
Cochrane Library	Alzheimer's disease, PET CT, neuroimaging, biomarkers, amyloid, disease progression, prognosis, diagnosis	Alzheimer's disease OR PET CT OR Neuroimaging OR Biomarkers OR Amyloid OR Disease progression OR prognosis OR diagnosis in Title Abstract Keyword	Last five years, neurology, mental health, diagnosis	263
Science Direct	Alzheimer’s disease, PET CT, biomarkers	"Alzheimer's disease" AND "PET CT" AND "Biomarkers"	Last five years, review articles, research articles, case reports, mini-reviews, English literature	457
Europe PubMed Central	Alzheimer's disease, PET CT, biomarkers, amyloid, disease progression, prognosis, diagnosis	"Alzheimer's disease" AND "PET CT" AND ("Biomarkers" OR "Amyloid") AND ("Prognosis" OR "Disease progression") AND Diagnosis”	Last five years, exclude preprints	352
Google Scholar	Alzheimer’s disease, PET-CT, plaque, amyloid, biomarkers, prognosis, disease progression, diagnosis	“(“Alzheimer’s disease” OR “dementia ”)” AND “(“PET CT” OR “Neuroimaging”) AND “(“Biomarkers”)” AND “(“Amyloid” OR “plaque, amyloid” OR “beta-amyloid peptides”)” AND “(“Disease progression” AND “Prognosis”)” AND “(Diagnosis” OR “Early diagnosis”)”	Last five years	1130

Inclusion and Exclusion Criteria

In this systematic review, we included papers published in the last five years, published in English with full text available, and based on human studies to reveal the most precise results. The included articles contained information regarding participants who underwent PET of the brain to diagnose AD biomarkers (Aβ amyloid, tau proteins, glucose metabolism, and synaptic density). Papers published more than five years ago, in foreign languages, and based on non-human studies were excluded to avoid bias and extract relevant information. Additionally, we excluded all conference abstracts, posters, and presentations that lacked full-text articles because of the limited availability of comprehensive data. Apart from the above, we excluded studies with patients who did not undergo PET to identify AD biomarkers.

Selection Process

All relevant articles were imported into EndNote (Clarivate, Philadelphia, PA), and all duplicates were removed. Each found article was screened by reading titles and abstracts. After identifying qualified articles, we assessed all selected papers for full-text availability. The most relevant papers were screened according to inclusion and exclusion criteria. Shortlisted articles that satisfy all criteria we included in the study.

Quality Appraisal and Process of Collecting Data

The appropriate quality appraisal tools were applied to the nominated studies according to their type of study. We used the Joanna Briggs Institute (JBI) critical appraisal methods to evaluate all selected cross-sectional research [19]. Additionally, we applied the Newcastle-Ottawa Scale to assess the cohort and longitudinal studies [20]. Two co-authors assessed the quality of the studies independently. After collecting all the relevant papers, each co-author contributed to the evaluation data for that systematic review. This study's primary outcome focused on the role of PET in the early diagnosis of AD by identifying biomarkers and benefits of PET in assessing AD progression and prognosis.

Results

**Figure 2:**
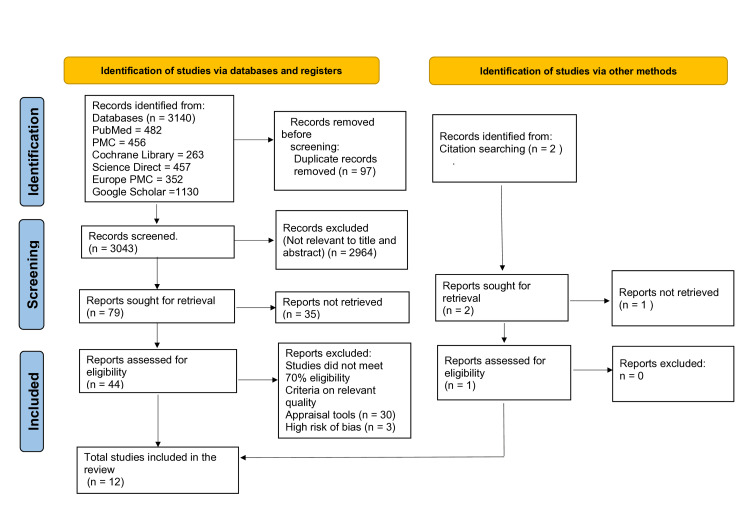
The PRISMA flowchart showing the article selection process. PRISMA: Preferred Reporting Items for Systematic Reviews and Meta-Analyses; PMC: PubMed Central.

The initial number of relevant studies identified on PubMed, PubMed Central, Europe PubMed Central, Science Direct, Cochrane Library, and Google Scholar was 3140. EndNote, in total, removed 97 duplicated articles. We reviewed 3043 articles in detail, screened through titles and abstracts, and assessed for full-text availability. As a result, 79 articles were shortlisted for retrieval, and 2964 papers were excluded. Shortlisted studies were evaluated using inclusion and exclusion criteria, and 35 articles were removed. In total, 44 relevant articles were assessed for eligibility and quality according to appropriate quality appraisal tools criteria. Additionally, two records were identified manually by citation, and one was not retrieved. One study was assessed for eligibility and was included in our review. As a result, 12 more relevant articles were included in the systematic review. We included articles with positive and negative results to avoid favoritism bias. The PRISMA flowchart, which illustrates the selection and screening process, is represented in Figure 2.

We used the Newcastle-Ottawa cohort checklist [20] to assess the quality of the eight included cohort and longitudinal studies. Table 2 demonstrates the assessment criteria.

**Table 2:** Newcastle-Ottawa cohort checklist used for quality assessment of the included non-randomized cohort and longitudinal studies. According to the Newcastle-Ottawa cohort checklist, a study receives one star “*” for questions 2 and 4 in the selection category and question 7 in the outcome category. Studies can obtain two stars “**” in questions 1 and 3 in the selection category, in question 5 in the comparability category, and in questions 6 and 8 in the outcomes category if the paper satisfies questionnaire requirements. To be considered high quality, the study requires three to four stars in the selection category, one or two stars in the comparability category, and two to three stars in the outcome. A study of fair quality would have two or three stars on selection, one or two stars on comparability, and two to three stars in the outcome category. Poor quality is indicated by zero to one star in selection, zero in comparability, and zero to one star in outcome category. Q1: representativeness of the exposed cohort; Q2: selection of non-exposure cohort; Q3: ascertainment of exposure; Q4: demonstration that outcome of interest was not present at the start of the study; Q5: comparability of the cohort based on the design or analysis; Q6: assessment of outcome; Q7: was follow-up long enough for outcomes to occur; Q8: adequacy of follow-up of cohorts.

Authors	Study design	Selection				Comparability	Outcome			Quality
		Q1	Q2	Q3	Q4	Q5	Q6	Q7	Q8	
Bullich et al. (2021) [12]	Non-randomized cohort	*	*	*	*	*	**		*	High
Zhang et al. (2024) [21]	Longitudinal study	*	*	*	*	*	**	*	*	High
Collij et al. (2023) [22]	Non-randomized cohort	*	*	*	*	*	**	*		High
Ackley et al. (2021) [23]	Non-randomized cohort	*		*	*	**	**	*	*	High
Librizzi et al. (2021) [24]	Non-randomized cohort	*		*	*	*	**			High
La Joie et al. (2020) [25]	Longitudinal study	*		*	*	*	**		*	High
Sintini et al. (2019) [26]	Longitudinal study	*		*	*	*	**		*	High
Chun et al. (2022) [27]	Longitudinal Study	*		*	*	*	**		*	High
Strobel et al. (2024) [28]	Non-randomized cohort	*	*	*	*	*	**		*	High

We used the JBI tool [19] to assess the quality of the four included cross-sectional studies. Two authors independently evaluated the risk of bias. A third reviewer assessed the results of the assessment. According to the quality appraisal tool results, studies were categorized as high, medium, and low quality. If a study obtains a score higher than 70%, it is classified as having high quality, studies with a score between 50% and 70% are classified as having medium quality, and articles with a score less than 50% are classified as having low quality. Studies that obtained scores higher than 70% were included in the review. Table 3 demonstrates the assessment criteria.

**Table 3:** The quality appraisal Joanna Briggs Institute (JBI) tool for cross-sectional studies. According to the Joanna Briggs Institute (JBI), quality appraisal tool questions studies able to obtain "Yes" if the study meets the criteria of assessment, "No" if the study does not meet criteria, "?" if the information is unclear, and "N/A" if the information is not applicable.

JBI critical appraisal	Ali et al. (2022) [29]	O'Dell et al. (2023) [30]	O'Dell et al. (2021) [31]
Were the criteria for inclusion in the sample clearly defined?	Yes	?	Yes
Were the study subjects and the setting described in detail?	Yes	Yes	Yes
Was the exposure measured in a valid and reliable way?	Yes	Yes	Yes
Were objective, standard criteria used for measurement of the condition?	Yes	Yes	Yes
Were confounding factors identified?	Yes	No	Yes
Were strategies to deal with confounding factors stated?	Yes	Yes	?
Were the outcomes measured in a valid and reliable way?	Yes	Yes	Yes
Was appropriate statistical analysis used?	Yes	Yes	Yes
Total score	8/8 (100%)	6/8 (75%)	7/8 (87.5%)
Results	Included	Included	Included

Study Characteristics

**Figure 3:**
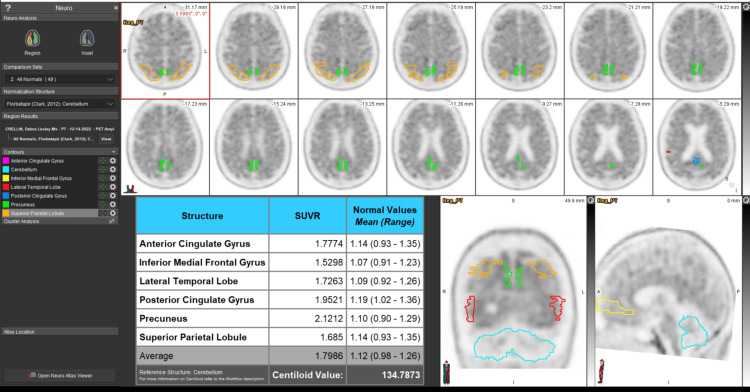
Pathological SUVR of tracer in the cingulate, medial frontal, lateral temporal, precuneus, and superior parietal lobes, which demonstrates the pattern of deposition of Aβ amyloid. SUVR: standardized uptake value ratio. The image was published with the permission of the original publisher and with a Creative Commons License. Image credits: Case courtesy of Dr. Sally Ayesa, Radiopaedia.org, rID: 166985.

**Figure 4:**
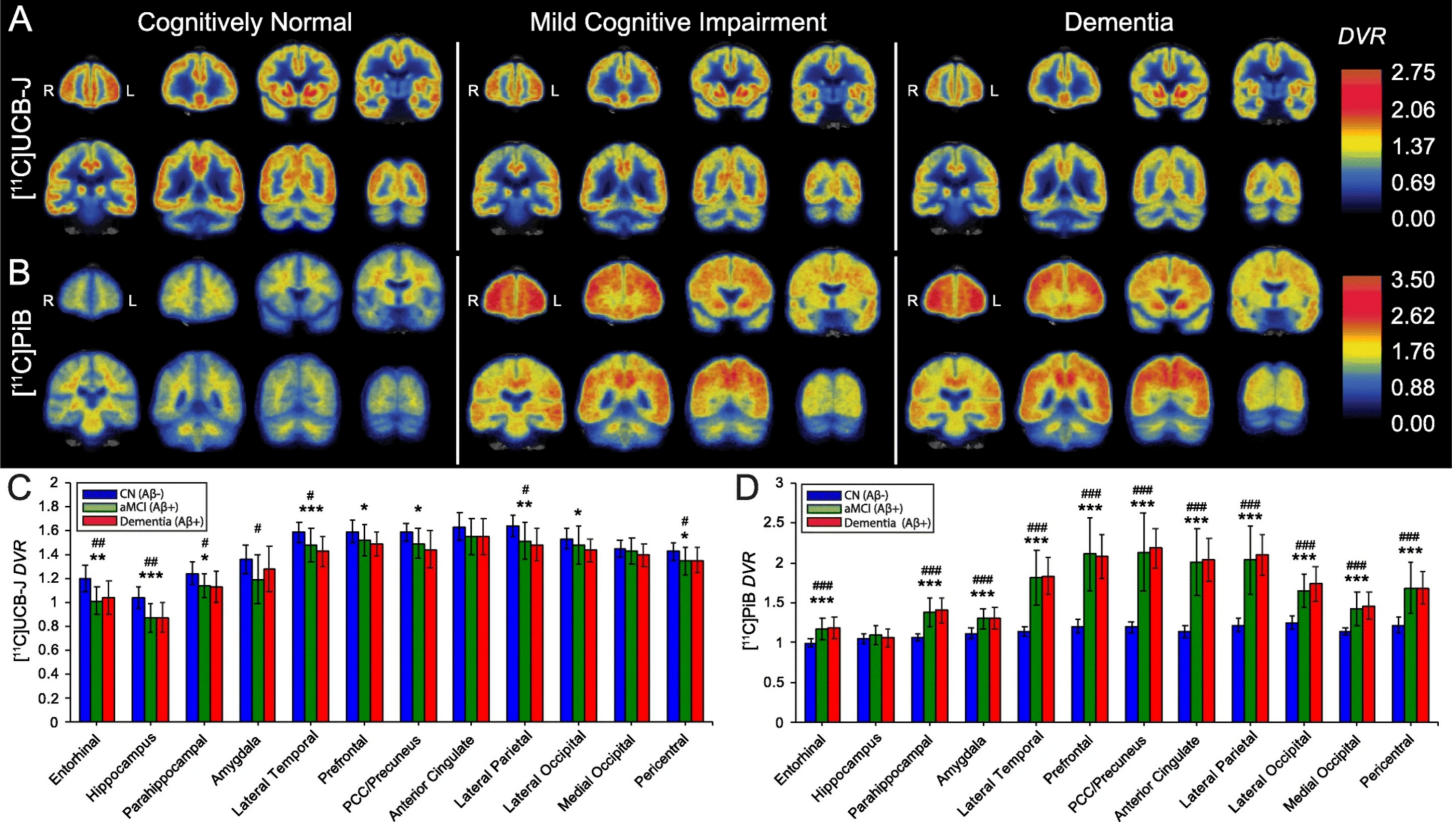
Deposition of [11C]UCB-J and [11C]PiB in PET demonstrates SV2A quantity and Aβ-amyloid deposition in the brain tissue. PET of [11C]PiB bio-tracer deposition, which binds Aβ-amyloid, and [11C]UCB-J, which binds synaptic vesicle glycoprotein 2A (SV2A). SV2A positively correlated to synapse density. Aβ-amyloid deposition significantly negatively correlates to synaptic density. DVR: distribution volume ratio using a whole cerebellum reference region; PCC: posterior cingulate cortex; aMCI: amnestic mild cognitive impairment; CN: cognitively normal. This figure is a republished image from the included article (https://doi.org/10.1186/s13195-020-00742-y) [30]. Permission was obtained from the original publisher and journal under the Creative Commons Attribution v4.0 International license (CC BY).

As a result of the selection and quality appraisal process, we included 12 relevant articles. All five non-randomized cohorts [11,22-24,28], four longitudinal studies [21,25-27], and three cross-sectional studies [29-31] are classified as having high quality. As a result, 2227 males and females participated in this systematic review. According to the results of our evaluation, the detection of Aβ amyloid PET significantly improved the early diagnosis of AD. The detection of tau protein demonstrated a significant potential for estimating AD's clinical and histological progression in longitudinal investigations. Table 4 below describes the articles retained in our review and their corresponding results (Figures 3, 4).

**Table 4:** The summary of the finalized articles. AD: Alzheimer’s disease; MCI: moderate cognitive impaired; CU: cognitively unimpaired; CN: cognitively normal; SUVR: standardized uptake value ratios; PCC: posterior cerebral cortex; PET: positron emission tomography; MMSE: Mini-Mental-State Examination; CDR-SOB: The clinical dementia rating-sum of boxes; PC: principal component; SV2A: synaptic vesicle glycoprotein 2A; FTLD: frontotemporal lobar degeneration; ROI: region of interest; FDG: fluorodeoxyglucose; FBB: florbetaben; aMCI: amnestic mild cognitive impairment.

References	Year of publication	Study design	Participants (n)	Results	Outcomes/conclusions
Bullich et al. [12]	2021	Cohort	686	The first pathological tracer retention areas were the cingulate cortices and the precuneus, frontal, inferior, and lateral temporal cortices. The sample of abnormal deposition (SUVR) of bio tracer, which binds Aβ-amyloid, is illustrated in Figure 4. The study showed that participants with established Aβ pathology or those in the "gray zone" significantly collected amyloid than subjects who tested negative for Aβ, and Aβ-negative or gray zone patients did not develop AD after a 4-year clinical follow-up. Among MCI participants, 91% had established Aβ pathology. Tau deposition was rare in those without a history of preexisting Aβ disease.	The results of this study improved the possibility of predicting the development of dementia during the MCI stage by measuring various amyloid loading, especially low levels, which can improve early diagnosis.
Zhang et al. [21]	2024	Non-randomized cohort	849	Results of the study showed that in the MCI and AD groups, the protein tau-181 deposition was significantly higher than in the CU group (p < 0.001). In the AD group, the deposition of protein tau-181 was higher than in the MCI group (p < 0.05).	Posterior cingulate cortex (PCC) SUVR detection allows for the prediction of the development and progression of dementia to AD. Additionally, glucose metabolism assessment significantly influenced PCC's capacity to predict AD development. The results of this study are essential for early diagnosis and theoretical justification for the therapeutic early prevention of AD.
Collij et al. [22]	2023	Longitudinal study	200	The results of this study showed that most amyloid-positive patients (122/128) were diagnosed with AD (95.3%). In patients with amyloid-negative results, AD was diagnosed in 40.2% (30/72). A postmortem pathological study of the brains of two patients revealed amyloid deposition and confirmed the diagnosis of AD.	The study's unique results on survival rate, cognitive deterioration, etiological diagnosis stability, and postmortem confirmation over an extended period show that amyloid-PET has clinical validity.
Ackley et al. [23]	2021	Cohort	154	When amyloid positive was added, the memory and executive function models showed better predictions of future cognition; the benefits were more significant for the executive function predictions.	Compared to models based solely on demographics and longitudinal cognitive evaluations, the inclusion of amyloid burden in this cohort marginally improved predictions of executive function measures and memory. These data may suggest that amyloid-PET does not significantly improve the predictive power of cognitive deterioration in environments where cognitive evaluation of cognitively normal patients is routine.
Librizzi et al. [24]	2021	Non-randomized cohort	40	The result of the study showed that patients who had positive 18F-florbetaben ((18F)FBB+) had lower scores on Mini-Mental-State Examination (MMSE) than patients with negative (18F)FBB− results.	The cerebral hypometabolism identified by (18F)FDG PET is beneficial for disease identification and staging if recorded CSF biomarkers and regularly checked MMSE values are insufficiently decisive despite the lack of pathological specificity. Additionally, a multi-biomarker approach, such as detecting tau protein and Aβ amyloid in the CSF, PET FDG, and PET FBB, is beneficial for precise AD diagnosis.
La Joie et al. [25]	2020	Longitudinal study	32	The study showed that the tau-PET signal predicted brain atrophy. Young patients showed a more essential relationship between brain atrophy and tau protein deposition.	The study's results revealed that tau protein deposition is a predictable pattern of neurodegeneration. Additionally, tau-PET can serve as a precise medicine tool to aid in predicting a patient's course and planning upcoming therapeutic trials.
Sintini et al. [26]	2019	Longitudinal study	30	The study's results revealed the negative impact of age on the clinical presentation of disease and tau protein deposition. The pattern of tau protein deposition was faster in younger individuals. High rates of tau accumulation were revealed in frontal and sensorimotor regions. Cortical atrophy and baseline volume correlated in the occipitoparietal areas. Higher atrophy rates and correlation with higher tau accumulation were revealed in the frontal and occipital lobes.	Tau protein accumulation in brain tissue and atrophy are regional. Tau protein deposition was in the frontal lobes, and brain atrophy was found in the temporal and occipital cortex. The results suggest a temporal discrepancy between neurodegeneration and protein deposition. Also, the study showed a positive association between brain atrophy rates and tau accumulation.
Chun et al. [27]	2022	Longitudinal study	25	The clinical dementia rating-sum of boxes (CDR-SOB) scores of the 18F-THK5351-positive group decreased more quickly than those of the 18F-THK5351-negative group (B = 0.003, p = 0.033).	The current study's findings imply that elevated 18F-THK5351 uptake, which may be linked to enhanced neuroinflammation, may be a valuable predictor of a poor prognosis among Aβ-aMCI patients.
Strobel et al. [28]	2024	Non-randomized cohort	24	PET imaging of [11C]PBB3 bio tracer deposition is beneficial for differential diagnosis between AD and frontotemporal lobar degeneration (FTLD). Accumulation of bio tracers [11C]PBB3 and [11C]PiB was significantly higher in patients with AD than in FTLD and strongly positively correlated to tau protein deposition. Additionally, [18F]FDG SUVR was reduced in the temporal and parietal lobes of participants with AD. Detection in CSF of tau protein and Aβ amyloid did not correlate with SUVR of bio tracers.	PET imaging of the brain using [11C]PBB3 has significant results in differentiation between AD and FTLD and strongly correlates with the deposition of tau protein and hypometabolism of glucose in the brain tissue. Apart from the above, cognitive assessment of patients revealed a negative correlation between [11C]PBB3 SUVR and results of MMSE in various areas of the brain, which suggests that tau protein deposition correlates with cognitive impairment. Early differential diagnosis between another neurodegenerative disorder, such as FTLD, could improve early AD diagnosis and appropriate management of disorders.
Ali et al. [29]	2023	Cross-sectional	99	This study showed higher regional SUVR (p < 0.05) in the posterior cingulate and precuneus lobes, and the result was significantly correlated with the deterioration of executive function. In contrast, other brain areas had no discernible correlation between memory and Aβ-PET SUVR.	According to the study, posterior cingulate regions are affected by Aβ pathology in the early stages. Alteration of executive functions can be revealed before memory deterioration in AD.
O'Dell et al. [30]	2023	Cross-sectional	64	Principal component (PC) subject scores of synapse loss were favorably linked with performance in all cognitive domains within the AD group, with positive loadings contributing similarly across most ROIs (Pearson r = 0.24–0.40, P = 0.06–0.006). Aβ-amyloid deposition was significantly negatively correlated to synaptic density, which was demonstrated by bio-tracer depositions ([11C]PiB PET binds Aβ-amyloid, and [11C]UCB-J binds synaptic vesicle glycoprotein 2A (SV2A)). Illustration of PET [11C] PiB PET and [11C]UCB-J deposition demonstrated in Figure 4.	According to this research, synaptic density is a reliable indicator of disease existence and severity in the early stages of AD. Aβ-amyloid deposition significantly negatively correlates to synaptic density.
O’Dell et al. [31]	2021	Cross-sectional	24	Global Aβ deposition in the hippocampus was significantly inversely correlated in individuals with MCI (r = -0.55, P = 0.04). In participants with mild dementia, there was no significant association (r = 0.05, P = 0.82; Fisher z = -1.80, P = 0.04). Regional SV2A binding and global Aβ deposition were not shown to be correlated in either diagnostic group, according to whole brain and exploratory studies conducted across other ROIs. ROI-based investigations of the relationship between SV2A binding and regional Aβ deposition did not show a consistent pattern.	Measures of hippocampal synaptic density and global Aβ deposition were found to be significantly inversely correlated in individuals with MCI but not dementia. Aβ may eventually decouple from neurodegenerative processes like synapse loss and reach a relative plateau consisting mainly of insoluble fibrillar Aβ, even while it still builds up in the early stages of clinical disease.

Discussion

Many studies have been published on early diagnosis of AD and PET detection of biomarkers, and some longitudinal clinical studies assessed the clinical progression of the disease and prognosis. This systematic review showed that PET imaging of AD biomarkers significantly improved the timely diagnosis of AD and allowed the choice of appropriate management and treatment plans. In addition, the authors mentioned the limitations of this study.

The Role of PET-CT in Early Diagnosis of Alzheimer’s Disease

**Figure 5:**
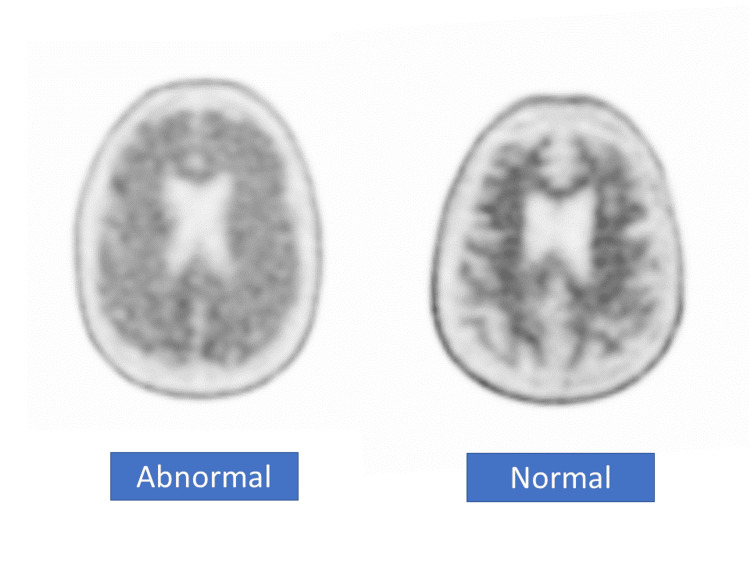
Generalized cortical amyloid deposition in the brain tissue. This picture demonstrates a pattern of significantly abnormal cortical amyloid deposition, which is consistent with Alzheimer's disease. The image was published with the permission of the original publisher and with a Creative Commons License. Image credits: Case courtesy of Dr. Sally Ayesa, Radiopaedia.org, rID: 166985.

According to this systematic review, PET imaging Aβ amyloid biomarker load is a highly specific biomarker, which in the medial cognitive impairment stage, even in low quantity, can predict progression to AD [11]. One of the included papers showed that early preclinical decline in AD was significantly correlated with the regional deposition of amyloid in the precuneus and posterior cingulate gyri, which was predictable to generalized brain amyloid deposition. The association was independent of the bio tracer SUVR level [29]. Another recent study demonstrated that Aβ amyloid deposition was consistently higher in all neocortical areas in both amnestic mild cognitive impairment (aMCI) and dementia patients, except for the hippocampus. It is also essential to consider that Aβ is intensively accumulating in the early phases of clinical disease and gradually gets closer to a relative plateau in the advanced stages [31]. Apart from the above, the study revealed a significant correlation between high accumulation of Aβ-amyloid in the precuneus and verbal impairment among patients with AD, which would predict memory deterioration in the preclinical stage of AD [29]. A picture of generalized brain amyloid deposition is illustrated in Figure 5.

Apart from the above, tau accumulation demonstrated a gradual pattern relative to disease progression, and in advanced stages, most tau uptake was registered in the frontal lobe. Among CU participants, significant tau depositions were detected in the hippocampus and middle frontal gyri, right inferior temporal lobes, and right posterior cingulate [21]. Additionally, age was negatively correlated to tau uptake in patients with atypical AD, and the most significant tau deposition was visualized in the frontal lobe. These findings would be beneficial for managing this group of patients [26]. Apart from the above, our findings suggest that PET imaging of tau-protein using [11C]PBB3 biotracer is beneficial for differential diagnosis between AD and another neurodegenerative disorder with overlapping symptoms, such as frontotemporal lobar degeneration (FTLD), even in the early stages [28].

In contrast, another included study demonstrated that glucose metabolism in the posterior cerebral cortex (PCC) significantly influences the ability to predict AD and has a great potential to be a prospective biomarker for early diagnosis of AD in CU patients. Results of the study revealed that glucose metabolism in PCC was positively correlated to the deposition of Aβ-amyloid, and glucose metabolism could be used as a complement to PET Aβ-amyloid and tau protein detection. These findings aid in the understanding of AD's early pathogenesis and offer theoretical justification for therapeutically preventing AD at an early age [21]. Additionally, the study showed that PCC SUVR of tracer has a high potential for predicting whether CU patients will progress to dementia and AD [21].

Additionally, another recent study revealed that the measure of spatial covariance of synaptic density correlates to neural tissue atrophy and can be beneficial for the prediction of the development of AD in CU and MCI patients [30,31]. Synaptic density and metabolism in AD show distinct regional synapse loss and hypometabolism patterns and were strongly negatively correlated to Aβ-amyloid deposition. Results of the study revealed that synaptic density is one of the most potent biomarkers of AD presence and severity. Postmortem study data confirmed that synaptic density is a reliable biomarker of memory impairment in patients with AD [30]. Apart from the above, using a combination of multiple biomarkers, such as amyloid-PET, tau-PET visualization, and CSF biomarkers, is more informative and increases the precision of diagnosis [24].

Alzheimer’s Disease Progression Assessment

The findings of this systematic review reported that amyloid-positivity was linked to low cognitive assessment performance in MMSE [22]. Apart from the above, the determination of amyloid-PET imaging allows for a precise diagnosis and prediction of the development of cognitive impairment from a long-term perspective [23]. A postmortem study proved the amyloid-positivity of patients and amyloid-negativity among patients without cognitive impairment and deposition of Aβ in PET [22]. Therefore, early diagnosis of tau positivity using PET results can increase the certainty of diagnosing dementia patients and allow the timely administration of symptomatic and disease-modifying treatment [24].

According to another recent study, the global intensity of the tau-PET signal predicted the rate of future atrophy regardless of baseline cortical thickness, according to quantitative analysis [25]. Further research revealed that the link between baseline tau-PET and subsequent atrophy was especially significant in younger patients and that the unique distribution of the tau-PET signal was a powerful indication of the topography of future atrophy [25,26]. Apart from the above, the longitudinal studies showed that atrophy still occurs in the areas with the most significant baseline changes and the fastest degeneration and atrophy rate in the occipital and parietal lobes due to the lowest baseline volumes. In contrast, in advanced stages of the disease, protein tau accumulation has shifted significantly to the frontal lobe. Similar results on distinct longitudinal spreading patterns for atrophy (more posterior) and tau uptake (more frontal) were observed in typical AD patients. Considering these geographical correlations between tau-PET uptake and atrophy is crucial, as it strengthens tau-PET's ability to predict neurodegeneration in specific brain regions, diagnostic impact, and development and assessment of future disease-modifying therapies [26].

In addition to demonstrating the value of tau-PET as a precision medicine tool to assist in predicting a patient's course and planning future clinical trials, these data support illness clinical models in which tau pathology is a key cause of local neurodegeneration [25]. PET diagnostic imaging of tau protein using [11C]PBB3 and [18F] fluorodeoxyglucose (FDG) bio tracers allows the study and comprehension of the pathology of neurodegenerative diseases, including AD, which has a high potential for monitoring AD progression [28].

Alzheimer’s Disease Prognosis

Regarding AD prognosis, our systematic review revealed that the only variable that significantly predicted mortality was diagnosis; patients with dementia had a greater chance of lethargic outcome at follow-up than patients without dementia. Apart from the above, the cognitive trajectory of patients with aMCI who test positive for THK5351-positive Aβ-18F presented that 18F-THK5351 was positive in 40.0% of Aβ-aMCI patients. Additionally, MCI patients with positivity for 18F-THK5351 tracer accumulation had a worse cognitive trajectory than patients with negative results for 18F-THK5351 enhancement. The current data considers that 18F-THK5351 positivity, which may be linked to elevated neuroinflammation, may be a valuable predictor of a poor outcome among Aβ-aMCI patients [27].

Future Research Directions

According to this systematic review, additional research is required to fully comprehend the temporal evolution of domain-specific cognitive change concerning the progression of amyloid deposition in different brain areas and to demonstrate this early preclinical amyloid change's longitudinal clinical and cognitive outcome [29]. In the future, it is necessary to perform cohort studies with a significant sample size to examine the correlation between synaptic loss and Aβ deposition and to analyze in vivo neuroimaging outcomes, like tau-PET or MRI [23,31]. The study should start in the preclinical stages of AD and observe it longitudinally in complex with other pathogenesis markers [31]. More studies are required to assess synaptic density pattern expression scores to forecast disease progression along the AD spectrum, including preclinical phases of AD pathogenesis. The association between synaptic density and other pathogenic markers, such as tau, amyloid, and FDG-PET, might be examined using additional data-driven techniques such as parallel independent component analysis or multimodal canonical correlation analysis [30]. According to this diagnostic investigation, more clinical studies are necessary to assess and improve therapy and assessment of AD [24].

Limitations

This systematic review thoroughly followed PRISMA 2020 guidelines and showed significant findings on the role of PET in diagnosing AD. The study includes a combination of recent relevant cohort, longitudinal, and cross-sectional studies with high quality, which revealed statistically significant results emphasizing the benefits of PET imaging of biomarkers that offer high specificity and sensitivity in predicting AD progression, cognitive decline, neurodegeneration and prognosis and the contribution of this method in early intervention. However, the study has several limitations that need to be described. The first limitation was including studies with small sample sizes and short follow-up periods. In the future, studies with large sample sizes and long follow-up periods are necessary [21,24,25,28,31]. The second limitation of this study was the restriction of clinical outcomes and length of follow-up periods based on mortality rate and other clinical circumstances, such as admission of patients to hospice or change of patient management plans [24]. Another limitation that could impact our results was false positive results in CU patients and diagnosis of AD and false negative results in patients with AD [27]. Apart from the above, we included only observational studies, which could be limited in establishing causality and effect relationships. Additionally, we performed a comprehensive search of literature through databases and included papers published in the last five years to find more recent papers to create precious results. Therefore, we could exclude older papers with potentially relevant findings.

## Conclusions

According to this systematic review, the detection of AD biomarkers using PET demonstrated significant results in early diagnosis, prognosis, and assessment of the progression of AD, which is essential for timely therapeutic interventions to slow or prevent AD progression before the development of severe cognitive decline. PET detection of Aβ amyloid is sensitive for detecting early amyloid accumulation, which predicts the onset of AD in both dementia-free and MCI patients, and results were correlated with linguistic difficulties and cognitive impairment. In contrast to amyloid-PET, visualization of increased tau protein accumulation strongly predicts future neurodegeneration and correlates with future brain tissue atrophy, which can predict prognosis. Additionally, PET imaging of AD biomarkers contributes to precision medicine, demonstrating a high potential for differential diagnosis between AD and other degenerative diseases with overlapping clinical presentations, which allows early intervention. Additionally, multiple biomarker approaches such as amyloid-PET, tau-PET visualization, and CSF biomarkers benefit AD diagnosis, prognosis, and treatment assessment.
